# Mobile GPU-based implementation of automatic analysis method for long-term ECG

**DOI:** 10.1186/s12938-018-0487-3

**Published:** 2018-05-03

**Authors:** Xiaomao Fan, Qihang Yao, Ye Li, Runge Chen, Yunpeng Cai

**Affiliations:** 1 Shenzhen Institutes of Advanced Technology, Chinese Academy of Sciences, Shenzhen, China; 2 University of Chinese Academy of Sciences, Beijing, China; 3 Shenzhen Engineering Lab for Health Big Data Analytic Technologies, Shenzhen, China; 4 Key Lab for Health Informatics, Chinese Academy of Sciences, Shenzhen, China

**Keywords:** Automatic ECG analysis, Parallel computing, Mobile GPU, Energy consumption

## Abstract

**Background:**

Long-term electrocardiogram (ECG) is one of the important diagnostic assistant approaches in capturing intermittent cardiac arrhythmias. Combination of miniaturized wearable holters and healthcare platforms enable people to have their cardiac condition monitored at home. The high computational burden created by concurrent processing of numerous holter data poses a serious challenge to the healthcare platform. An alternative solution is to shift the analysis tasks from healthcare platforms to the mobile computing devices. However, long-term ECG data processing is quite time consuming due to the limited computation power of the mobile central unit processor (CPU).

**Methods:**

This paper aimed to propose a novel parallel automatic ECG analysis algorithm which exploited the mobile graphics processing unit (GPU) to reduce the response time for processing long-term ECG data. By studying the architecture of the sequential automatic ECG analysis algorithm, we parallelized the time-consuming parts and reorganized the entire pipeline in the parallel algorithm to fully utilize the heterogeneous computing resources of CPU and GPU.

**Results:**

The experimental results showed that the average executing time of the proposed algorithm on a clinical long-term ECG dataset (duration 23.0 ± 1.0 h per signal) is 1.215 ± 0.140 s, which achieved an average speedup of 5.81 ± 0.39× without compromising analysis accuracy, comparing with the sequential algorithm. Meanwhile, the battery energy consumption of the automatic ECG analysis algorithm was reduced by 64.16%. Excluding energy consumption from data loading, 79.44% of the energy consumption could be saved, which alleviated the problem of limited battery working hours for mobile devices.

**Conclusion:**

The reduction of response time and battery energy consumption in ECG analysis not only bring better quality of experience to holter users, but also make it possible to use mobile devices as ECG terminals for healthcare professions such as physicians and health advisers, enabling them to inspect patient ECG recordings onsite efficiently without the need of a high-quality wide-area network environment.

## Background

With the development of information and healthcare technologies, large number of people tend to monitor their health condition at home using wearable holters. Due to the unique nature of healthcare service, users typically follow a similar schedule and upload the electrocardiogram (ECG) data collectively, creating a high computational burden on the healthcare platform at a certain time of day. In particular, concurrent processing of long-term ECG data, which is an important assistant approach to capture intermittent cardiac arrhythmias, tortures the healthcare platform heavily. How to efficiently process long-term ECG data collectively is an important issue to be solved.

ECG, as one of the vital physiological data, shows the time evolution of the heart’s electrical activity, which is caused by distinct electrical depolarization–repolarization patterns of the heart. Disorders of heart rate or rhythm, or changes in the morphological patterns, are indicators of underlying diseases [1]. For example, myocardial infarction, cardiomyopathy, and myocarditis can lead to obvious ECG changes. Various ECG heartbeat classification methods were proposed by researchers in previous studies [2, 3]. An automatic ECG heartbeat classification pipeline mainly consists of basic steps including noise reduction [4–6], QRS complex detection [7, 8], feature extraction [9–11], and heartbeat classification [3, 12–18]. Heartbeat classification is a critical step in automatic ECG analysis. In the past few years, heartbeat classification methods are still under development with novel algorithms being proposed continuously. Liang et al. used hidden markov models (HMMs) to classify patient ECG signals in the free-living environment [13]. Lannoy et al. proposed weighted conditional random fields classifier for the automatic classification of heartbeats [17]. Furthermore, with the development of machine learning, many artificial intelligent methods have been applied in ECG heartbeat classification. Ye et al. applied wavelet transform and independent component analysis separately to each heartbeat to extract morphological features, then support vector machine (SVM) was used for heartbeat classification [14]. Yu et al. used wavelet transformation and probabilistic neural network (PNN) to classify the ECG heartbeats [18]. Oliveira et al. employed a dynamic bayesian network to predict premature ventricular heartbeats [15]. Lagerholm applied unsupervised clustering methods to partition the QRS complex into clusters, then self-organized neural networks were used to identify heartbeat types [16]. However, most ECG heartbeat classification methods are time-consuming, especially for processing long-term ECGs such as 24-h long ECGs. Although healthcare cloud platforms have been widely built to collect and manage ECG data from large populations, it is technically infeasible to provide prompt feedback for a large number of concurrent ECG analysis requests for even a median platform managing ten thousands of users.

To reduce the burden of remote healthcare platforms, many researchers shifted analysis tasks from remote healthcare platforms to local mobile computing devices [19–21]. However, processing long-term ECG data on mobile computing devices is time-consuming and leads to poor quality of experience as it occupies too many central processor unit (CPU) computation resources which are limited on mobile devices. At the same time, the heavy computation demanded by long-term ECG analysis leads to a long period in which the CPU runs at full frequency, which greatly shortens the working hours of battery in case the ECG analyzing tasks are frequently launched, i.e., in a mobile device of a physician or a health advisor. With the aforementioned limitations of mobile devices, a fast and energy-efficient analysis algorithm for long-term ECG is urgently needed.

This paper aimed to propose a novel parallel automatic ECG analysis algorithm based on mobile graphics processing unit (GPU). The 24-h long ECG recordings collected from volunteers were utilized to evaluate the computing performance and energy efficiency of the parallel algorithm. The test was conducted on a smartphone named OnePlus 3 equipped with an Qualcomm Snapdragon 820 processor along with 6 GB memory and an integrated Adreno 530 GPU. The parallel automatic ECG analysis algorithm was optimized through workgroup size tuning, data vectorization, and zero memory copy technology to take full advantage of the potential of heterogeneous computation. Additionally, the optimized parallel automatic ECG analysis algorithm consumed less energy per second in average compared with the sequential automatic ECG analysis algorithm. This led to a significant energy saving and longer working hours of battery for mobile devices when they were served as ECG processing terminals. Note a preliminary version of this paper has been reported [22].

The subsequent sections of the paper are organized as follows. Section of “Methods” introduces the OpenCL parallel computing model, parallel optimization technologies, the sequential automatic ECG classification algorithm, and the parallel implementation of the sequential automatic ECG classification algorithm on mobile GPUs. The results including experimental environment, data source, classification performance of automatic ECG analysis algorithm, and parallel efficiency and energy efficiency are described in the section of “Results and discussion”. The final section summarizes the paper.

## Methods

### The OpenCL parallel computing model

**Fig. 1:**
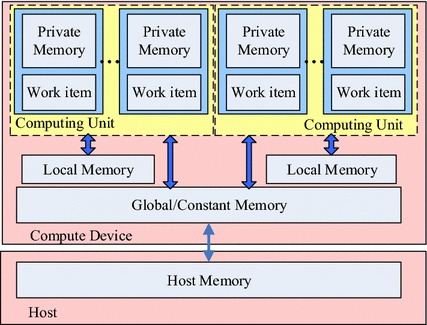
Memory model of OpenCL framework

OpenCL is a common multi-CPU/multi-GPU heterogeneous computing framework. OpenCL uses heterogeneous CPU/GPU hardwares to perform large-scale computation tasks, especially tasks with high parallelism, more efficiently taking full advantage of the powerful parallel GPU computation capabilities [23, 24]. OpenCL has many advantages, such as platform independence, simple hardware acceleration, and easy programming. Figure 1 shows the memory model of OpenCL framework. Memory hierarchy of a GPU device consists of global memory, constant memory, local memory, and private memory. Global memory and constant memory are shared among all computation units, while every computing unit owns independent local memory which can be accessed with much shorter latency. All work items in a workgroup share the same local memory. This model owns great generality and matches well with the computing architecture of mobile devices. Generally, OpenCL provides a unified computing model that allows many intensive tasks of mobile computation to be implemented in parallel and assigned to mobile GPU. It shortens the executing time and proffers users better quality of experience.

### Automatic ECG analysis algorithm

**Fig. 2:**
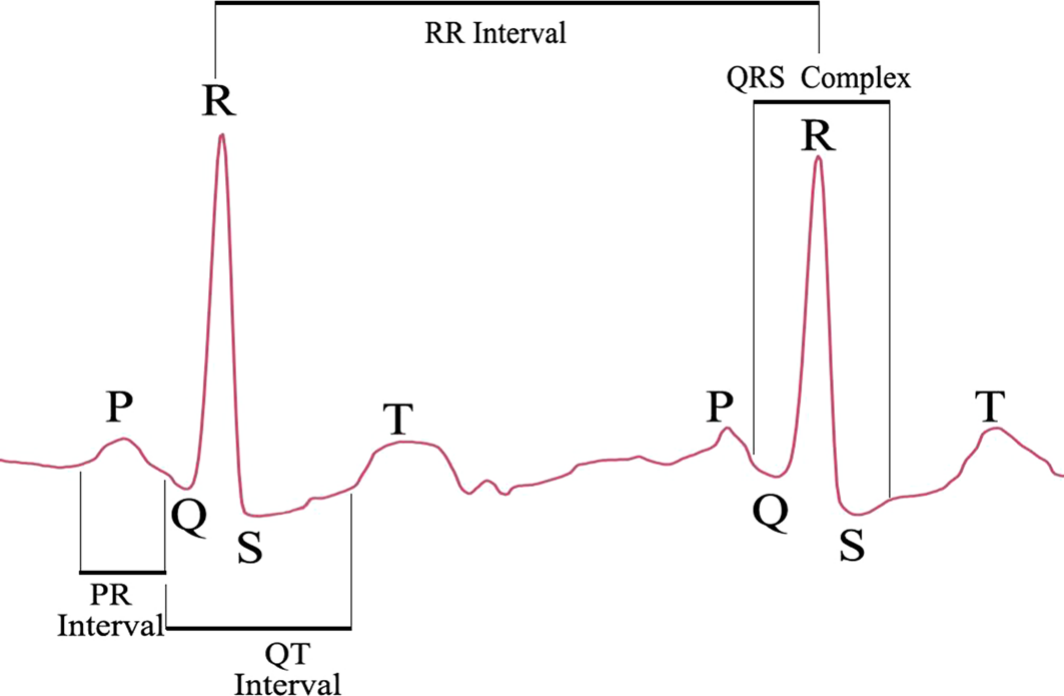
ECG signal example with two cycles

ECG is an electrical signal caused by the electro-physiologic activities of the human heart. A normal heartbeat of ECG includes the P, Q, R, S, and T wave segments. As shown in Fig. 2, two prominent features of ECG are the RR interval and QRS complex width. The RR interval is the width between two nearby R peaks, and the QRS complex width is defined as the distance between Q and S wave. Other features of ECG include PR interval, QT interval, ST segment, and T wave. Accurate identification of ECG features is important for the automatic ECG analysis algorithm.

**Fig. 3:**
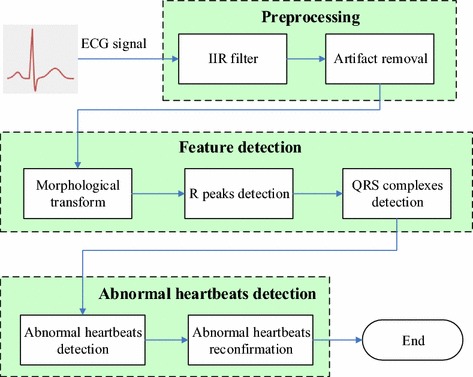
Flowchart of the automatic ECG classification algorithm

Figure 3 shows the entire computation procedure of the automatic ECG analysis algorithm, which consists of three processing phases: (1) preprocessing phase; (2) feature detection phase; (3) abnormal heartbeat detection phase. The preprocessing phase includes two steps: infinite impulse response (IIR) band-pass filtering and artifact removal. The IIR bandpass filter with a cut-off frequency of 0.5 and 40 Hz is used to cancel baseline drift, high-frequency noise, and powerline interference [25]. Then, in the artifact removal step, the dynamic threshold method is used to mark the noise signal segments that would be abandoned in the succeeding processes [26]. In the feature detection phase, the morphological transform method is used to enhance the amplitude of R peaks to locate the positions of R peaks and QRS complexes [27]. R peak positions can be detected easily based on the dynamic threshold method. The start and end positions of QRS complex are set at 50 ms before R peak and 80 ms after R peak, respectively [2]. In addition, the RR interval and the QRS complex width are calculated. In the abnormal heartbeat detection phase, abnormal heartbeats are identified based on judgment rules given by physicians with the acquired position lists of R peaks and QRS, RR intervals, and the QRS complex width [29, 30]. Moreover, the template matching method is utilized to reconfirm abnormal heartbeats to avoid the misjudgment of abnormal heartbeats. Following general clinical ECG arrhythmia detection guidelines [28], this approach could identify five types of abnormal ECG arrhythmias, including normal sinus heartbeats (NB), bigeminy (BG), trigeminy (TG), atrial premature heartbeats (APB), and ventricular premature heartbeats (VPB). The detailed judgments of 5 types of ECG rhythms are listed in Table 1.

**Table 1:** Detailed judgment rules of 5 types of ECG rhythms.

Type of rhythms	Rules
NB	QRS complex width < 120 ms
	0.75× average RR interval < current RR interval
	Current RR interval < 1.2× average RR interval
APB	QRS complex width < 120 ms
	Current RR interval < 0.75× current average RR interval
	Current RR interval + next RR interval < 2× current average RR interval
VPB	QRS complex width > 120 ms
	Current RR interval < 0.75× current average RR interval
	Current RR interval + next RR interval ≥ 2× current average RR interval
BG	Alternating appearance of VPB and NB
TG	Alternating appearance of 2 NBs and 1 VPB

### Parallel implementation of automatic ECG classification method on OpenCL framework

**Fig. 4:**
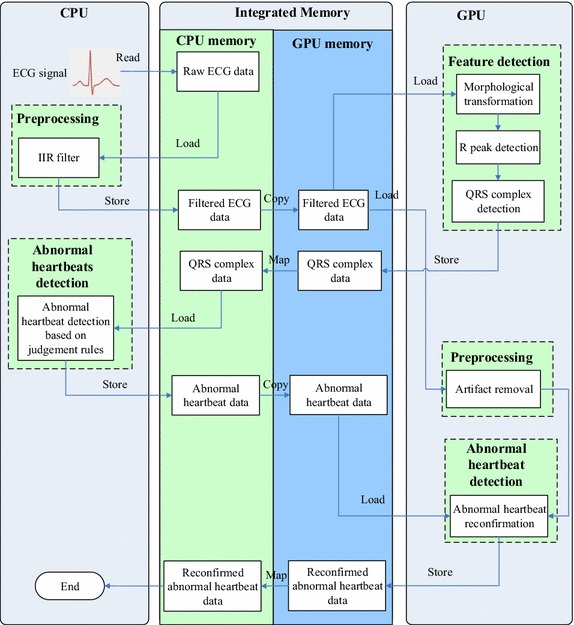
Architecture of the parallel automatic ECG analysis algorithm on smartphone-based GPU

Modern mobile devices have a highly integrated circuit that combines all the primary components, such as CPU, GPU, and memory, into a single chip. One of the benefits is high bandwidth memory. The ultra-wide memory standard essentially accelerates the data transfer speed between memory and CPU/GPU. Another important feature is that CPU and GPU memory are integrated on the same chip and separated by embedded software. Instead of repeatedly transferring data between CPU and GPU memory in accordance with the task shifting, memory mapping technology could be introduced to map the same piece of physical memory in both the memory space of CPU and GPU. As a result, data transfer can be cut down or even avoided. Figure 4 shows the entire architecture of the parallel automatic ECG analysis algorithm. Compared with the sequential algorithm, the entire program flow in parallel algorithm is reorganized in this study to fully utilize CPU/GPU heterogeneous computing resources. IIR filter is hard to be parallelized due to its tight-coupling pattern and a processing iteration relies on the result of the previous one [31]. Therefore, it remains running on CPU. The morphological transform method with inherent parallel characteristic is implemented in parallel on GPU. The following processes including R peak detection and QRS complex detection are also implemented in parallel on GPU. The abnormal heartbeat detection runs on CPU to better utilize CPU computing resources. At the same time, the artifact removal process is implemented in parallel on GPU. Finally, QRS complex templates are generated from noise-free signals and used to correct misjudgment on abnormal heartbeats. For details of the parallel implementation, refer to Algorithm 1.

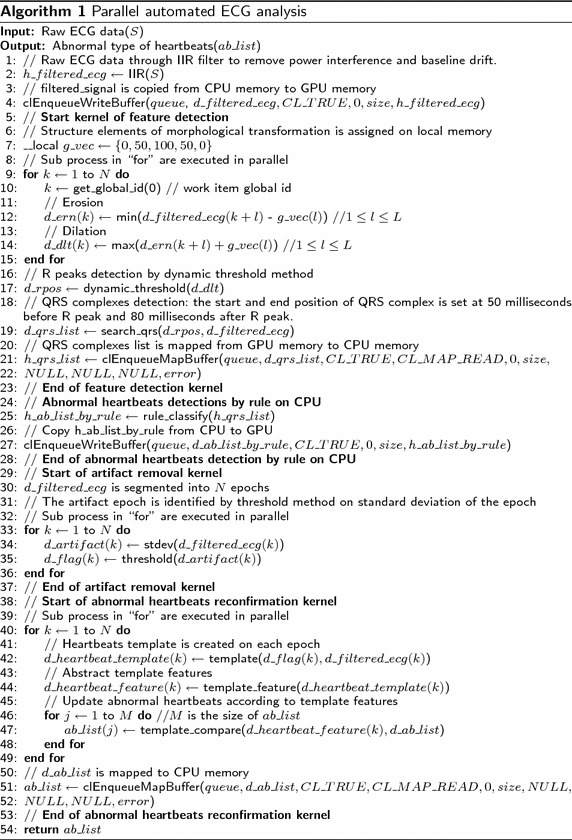



### Parallel optimization technologies

The parallel algorithm could obtain a higher speedup through workgroup size tuning, data vectorization, and zero memory copy technologies. The details as follows: (1) workgroup size tuning. Workgroup size is an important factor affecting program efficiency. No general formula is available to calculate the optimal workgroup size, besides some constraints. Larger workgroups are better at hiding memory latency, while they would lead to cache thrashing. Proper workgroup size must be determined considering the register usage and the presence of barrier instructions inside kernels. (2) Data vectorization technology. As vector operations are common in OpenCL framework, GPUs are designed to be particularly powerful in dealing with vectors. Native vector operations, including addition and multiplication, are supported by most of GPU architectures with the development of mobile GPU technologies. Meanwhile, GPU memory architecture has also been developed to be efficient in loading/storing batches of data concurrently. (3) Zero memory copy technology. Data transfer between CPU and GPU memory is quite slow. It is a common case that the time cost of pure computation in parallelized programs takes up only a small portion of the entire time cost. The intrinsic latency for data transfer between CPU and GPU memory is a big obstacle in parallelization practice on the traditional CPU/GPU heterogeneous architecture. The CPU and GPU in mobile devices are usually integrated in one chip owing to the compact hardware structure of smartphones, making it possible for them to share the same memory. The CPU can recognize the memory allocated by the GPU as a part of its own memory with the zero memory copy technology and access it directly. Instead of repeatedly copying data between CPU and GPU whenever needed, the algorithm spends only its efforts on the initial mapping that takes comparatively much lesser time.

In order to maximally utilize the computing resources, these technologies were applied on the proposed parallel program. For workgroup size, we started from 64, and scaled up and down until the peak performance was found. The result was that most tasks were most efficiently completed when workgroup size was 128, while a workgroup size of 256 offered the highest performance for the task morphological transformation, as they relied on less registers. For data vectorization, the data were loaded/processed/stored in batches of 128 bits for all the transactions. As 32-bit float-point numbers were employed, four numbers were packed as a vector and operations on them were re-written using vector instructions. For zero memory copy, it was utilized throughout the design of the entire parallel algorithm. When CPU requires processed data from GPU, data were mapped directly to host memory from GPU memory directly, as it was shown in Fig. 4.

## Experiments

### Experimental environment

The compiling and linking from Android application through Java and C/C++ to OpenCL could be complemented owing to the Java Native Interface designed to enable native libraries’ calls from Java code. The experimental environment is benchmarked on a smartphone named OnePlus 3 equipped with a Qualcomm Snapdragon 820 processor along with memory of 6 GB. In detail, the processor consists of two CPU cores running at 2.15 GHz and another two cores running at 1.6 GHz, together with an integrated Adreno 530 GPU in which 256 arithmetic and logic units run at 624 MHz.

### Data collection

In this study, a multi-parameter patient simulator (FLUKE ProSim 8), the heart rate of which was 60 beats/min, was used to generate 5 types of ECG signals. Then, our developed wearable Holter (Mini-Holter, 58 $\times$ 50 $\times$ 10 mm, 20 g, over 30  h of battery working time, single channel three leads, 150/250/500 Hz sampling frequency) was connected with the multi-parameter simulator via ECG lead wire cables to capture 40 5-min long ECG recordings at a sampling frequency of 150 Hz. A total of 3120 NB, 240 APB, 240 VPB, 600 BG and 400 TG were generated. These simulated ECG records were utilized to evaluate the classification performance of the automatic ECG analysis. On the other hand, a public ECG dataset named MIT-BIH arrhythmia database [32] (total 48 records including 74,695 NB, 2745 APB, and 5976 VPB) was also utilized for the classification performance. These ECG records were sampled at 360 Hz with 11-bit resolution over a 10 mV range. Heartbeat types of NB, APB and VPB were selected for identification in this paper.

To further verify the performance of the proposed method on clinical ECG records, seven subjects (2 males and 5 females) from the General Hospital of People’s Liberation Army (301 hospital) were recruited to generate independently representative dataset. The age of the subjects ranged from 49 to 75 years, reaching an average of 63 ± 9 years. Each subject wore the Mini-Holter (150 Hz sampling frequency) to collect a long-term ECG signal, the duration of which is 23 ± 1.04 h (ranging from 21.1 to 24.0 h). The clinical long-term ECG dataset consists of 673147 NB, 481 APB, 268 VPB, 2 BG, and 1 TG. All the subjects were requested to sign the informed consent form before the experiment. At the same time, abnormal arrhythmia heartbeats of the dataset were marked by an expert physician.

### Experimental procedure

**Fig. 5:**
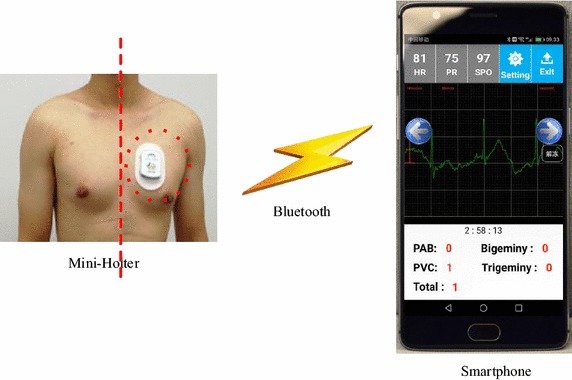
Experimental procedure. Long-term single lead ECG signals were taken by Mini-Holter. After the Mini-Holter was taken down from body, the ECG signals were transfer to smartphone for analysis via Bluetooth

As Fig. 5 shown, Mini-Holter was placed on the skin of volunteer’s chest with glue-like gel to record a long-term ECG signal. The attached position was between the middle line of the body and the heart. Volunteers would wear Mini-Holters for 12–24 h as they go about daily routine. After ECG data collection was completed, the Mini-Holter would be taken down from volunteer’s body. The long-term ECG data was transferred to smartphone via Bluetooth. The first and last 2 min of data were cut off to ensure that the signal is stable as volunteers need time to attach/detach the Mini-Holter. The data was analyzed by aforementioned mobile GPU-based automatic ECG analysis method. Then, subjects could browse the analysis results and original ECG signals. At the same time, the executing time of the program was recorded backstage as well as the energy consumption using Trepn Profiler from Qualcomm. Experiments of power consumption were conducted at a starting battery life of 100% or close to 100%. Both parallel and sequential program were executed five times on each tested ECG record and results were saved to be averaged. As for throttling, we launched a third-party CPU adjuster to keep CPU running at full frequency while the tested experiments were conducted.

## Results and discussion

### Classification performance

Several metrics including classification accuracy (*ACCU*), sensitivity (*SENS*), and specificity (*SPEC*) were calculated to measure the classification performance, and they are defined as follows combining statistical quantities:

1 $$\begin{aligned} ACCU = \dfrac{TPOS + TNEG}{TPOS + FNEG + FPOS + TNEG} \end{aligned}$$

2 $$\begin{aligned} SENS = \dfrac{TPOS}{TPOS + FNEG} \end{aligned}$$

3 $$\begin{aligned} SPEC = \dfrac{TNEG}{FPOS + TNEG} \end{aligned}$$

where *TPOS* is true positive, *TNEG* is true negative, *FPOS* is false positive, and *FNEG* is false negative.

The performance of the sequential and parallel algorithm in detecting APB, VPB, BG, TG, and NB was evaluated on the synthetic dataset. The experimental results were shown in Table 2. The accuracy, sensitivity and specificity were more than 98%. On the other hand, MIT-BIH arrhythmia database was also employed to evaluate the classification performance of NB, APB and VPB. The results were shown in Table 3. The sensitivity of the proposed algorithm in detecting APB and VPB were 86.12 and 89.01%. Compared with the noise-free synthetic data, the sensitivities of the proposed algorithm in detecting on MIT-BIH arrhythmia database decreased a little bit.

**Table 2:** Classification performance of the sequential and parallel automatic ECG algorithm on synthetic ECG data

Type of rhythms	Number of records	*SENS* (%)	*SPEC* (%)	*ACCU* (%)
NB	3120	98.92	99.52	99.02
APB	240	99.60	99.34	99.34
VPB	240	98.03	99.02	98.00
BG	600	99.33	98.77	98.81
TG	400	100	99.07	99.10
Average		99.18	99.14	98.85

**Table 3:** Classification performance of the sequential and parallel automatic ECG algorithm on MIT-BIH arrhythmia database

Type of rhythms	Number of records	*SENS* (%)	*SPEC* (%)	*ACCU* (%)
NB	74,695	98.24	97.76	98.15
APB	2745	86.12	99.27	99.03
VPB	5976	89.01	99.76	98.95
Average		91.12	98.93	98.71

Apart from validating the proposed method’s high classification performance on the synthetic dataset and MIT-BIH arrhythmia dataset, we also evaluated the performance on clinical ECG dataset which was collected from the 301 hospital. As shown in Table 4, over 94.53% of normal sinus heartbeats were identified. The sensitivity of APB and VPB were 80.04 and 83.95%, correspondingly. The classification performance of the proposed program in detecting APB and VPB on the clinical ECG data was obviously lower than that on the synthetic data. The cause was that clinical ECG data were collected by wearable Mini-Holter and contaminated by a variety of noise such as artifact noise, power frequency interference, baseline drift, etc. The signal-to-noise (SNR) of the noise-free synthetic data was obviously higher than that of the clinical ECG data. The number of subjects who suffered BG and TG is very little, two subjects (one suffered two events of BG in a day and another suffered one event of TG in a day) were found to satisfy our experimental requirement. The sensitivity, specificity, accuracy of BG and TG were 100% as two events of BG and one event of TG were correctly identified. It demonstrated that our proposed method could identify BG and TG correctly even if BG/TG are few in number.

**Table 4:** Classification performance of the sequential and parallel automatic ECG algorithm on clinical long-term ECG data

Type of rhythms	Number of records	$SENS$ (%)	$SPEC$ (%)	$ACCU$ (%)
NB	673,147	94.53	83.64	94.52
APB	481	80.04	96.31	96.30
VPB	268	83.95	98.88	98.87
BG	2	100	100	100
TG	1	100	100	100
Average		91.70	95.77	97.94

### Parallel program efficiency

In order to give users real-time response after their physiological data were transmitted to smartphones, it was considered only viable to run automatic ECG analysis on powerful servers. In this study, the focus was shifted from powerful computing servers to long-underestimated potential computing capability of smartphone-based GPU. Parallel structures in the sequential automatic ECG analysis algorithm were fully extracted, and then these specific parts were redesigned using a framework designed for computing heterogeneous platforms known as OpenCL.

Speedup is an effective measurement to validate the efficiency of the parallel program [33]. It is defined as the proportion of the elapsed time when executing a program on a single processor to the execution time on *n* processors. The speedup with *n* processors is defined as:

4 $$\begin{aligned} SP(n) = \dfrac{T_{s}}{T_{p}} \end{aligned}$$

where *SP*(*n*) is speedup with *n* processors. $T_{s}$ is the execution time of the algorithm running on a single processor. $T_{p}$ is the execution time of program running on *n* processors.

The time-consumption analysis of the automatic ECG algorithm was performed on a 24-h long ECG signal (no. 5) which collected from the 301 hospital. It was found that the artifact removal, feature detection, and abnormal heartbeats detection took up 16.7, 60.4, and 15.1% of the time cost, respectively, summing up to more than 92.2%. Therefore, the artifact removal, feature detection, and abnormal heartbeats detection were implemented in parallel with OpenCL framework to shorten the executing time of the entire automatic ECG analysis algorithm.

**Fig. 6:**
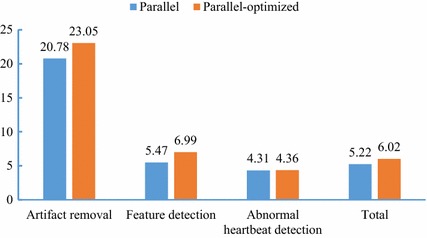
Speedup of the parallel automatic ECG analysis algorithm. Parallel means the parallel automatic ECG algorithm; parallel-optimized means that the parallel automatic ECG algorithm was further optimized by workgroup size tuning, data vectorization and zero memory copy technologies

The same 24-h long ECG signal was also selected to evaluate the computing efficiency of the parallel program in each step. As shown in Table 5 and Fig. 6, the time cost of the automatic ECG analysis algorithm was reduced from 7.57 to 1.45 s. The parallel automatic ECG analysis algorithm achieved a speedup of 5.22× compared with the sequential algorithm. Then the parallel algorithm was further optimized using workgroup size tuning, data vectorization and zero memory copy technologies. With the optimization mentioned above, the time cost of parallel algorithm was further reduced to 1.26 s, which achieved a speedup of 6.02×. In detail, the optimized parallel automatic ECG algorithm achieved a speedup of 23.05×, 6.99×, and 4.36× in the artifact removal, QRS complex detection, and waveform classification respectively. Meanwhile, the remained long-term ECG signals were also utilized to test the computing efficiency. As shown in Table 6, the average executing time of the sequential program and the parallel program on long-term ECG dataset (duration: 23.0 ± 1.0 h) were 7.018 ± 0.592 and 1.215 ± 0.140 s correspondingly. The parallel program achieved an average speedups of 5.81 ± 0.39×, which showed its great superiority over the sequential algorithm.

**Fig. 7:**
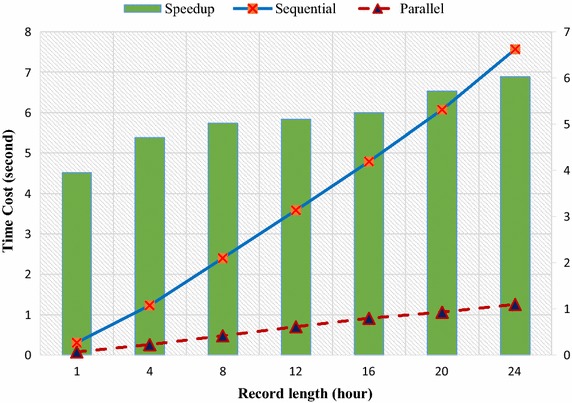
Time cost analysis of the parallel program with ECG records with different lengths. Parallel means parallel automatic ECG classification algorithm; sequential means sequential automatic ECG classification algorithm

To further explore the proposed parallel algorithm’s performance for records with different lengths, short signals, the duration which were 1, 4, 8, 12, 16, 20, and 24 h, were generated by randomly cropping a 24-h ECG signal. As shown in Fig. 7, both parallel and sequential algorithm presented a near-linear execution time increase in response to the increase of data length. More obviously presented by the speedup curve, was that the speedup increased sharply when data length increased from 1 to 4 h, and entered into a much gentle ascendance afterwards. Two factors would help to interpret this trend: (1) the utilization rate of computing resources dropped when input was too short, as the lack of workloads caused the tasks to be more sparsely assigned to the GPU. (2) Unavoidably, part of the algorithm contributed a constant part to the execution time, and became more significant as the other part shrank in accordance with the input size.

**Table 5:** Speedup of the parallel automatic ECG analysis algorithm in each step on a 24-h long ECG signal

Step	Sequential program (s)	Parallel program (s)	Parallel-optimized program (s)	Speedup (parallel vs. sequential)	Speedup (parallel-optimized vs. sequential)
Artifact removal	1.267	0.061	0.055	20.78×	23.05×
QRS complex detection	4.560	0.834	0.652	5.47×	6.99×
ECG waveform classification	1.155	0.268	0.265	4.31×	4.36×
Total	7.565	1.448	1.257	5.22×	6.02×

**Table 6:** Speedup of the parallel program on the long-term ECG dataset collected from the 301 hospital

ECG no.	Length (h)	Sequential program (s)	Parallel program (s)	Speedup
1	23.3	7.412	1.307	5.67×
2	22.9	5.926	0.935	6.34×
3	24.0	7.530	1.331	5.66×
4	21.1	6.984	1.125	6.21×
5	24.0	7.565	1.257	6.02×
6	22.2	6.594	1.255	5.26×
7	23.4	7.118	1.294	5.50×
Average	23.0 ± 1.0	7.018 ± 0.592	1.215 ± 0.140	5.81 ± 0.39×

### Program energy efficiency

The need for the computing power of smartphones has increased with the continuing trend for smartphones to be more versatile. However, the greater performance comes with a price, which includes the reduced working hours of the battery. Thus, energy consumption is one of the key performance indicators of an application. In order to evaluate the energy efficiency of the proposed algorithm, the long-term ECG dataset from the 301 hospital were also utilized for test. The average battery power and total battery usage of test smartphone were recorded with Trepn Profiler provided by Qualcomm, which was reported to have an accuracy of 99% when Monsoon Power Monitor was used as a reference [34]. Furthermore, compared with the sequential program, the parallel program could save energy consumption greatly. The energy conservation is defined as:

5 $$\begin{aligned} E_{save} = \dfrac{E_{s} - E_{p}}{E_{s}} \end{aligned}$$

where $E_{save}$ is the energy saved by the parallel program, $E_{s}$ is the energy consumption of the sequential program, and $E_{p}$ is the energy consumption of the parallel program.

As shown in Table 7, the average power of the sequential algorithm was 1860.95 ± 42.07 mW, while the average power of the parallel algorithm was 1826.91 ± 21.44 mW. The parallel automatic ECG analysis algorithm consumed less power per second in average compared with the sequential algorithm. The average battery energy consumption of the sequential algorithm executed five times was 33.93 ± 1.95 mWh, while the parallel algorithm executed five times consumed 12.16 ± 0.65 mWh. According to Eq. (5), the parallel algorithm could save about 64.16% energy. However, the energy consumption was recorded for the whole Android application, which also included data loading and other operations, as it was very hard to measure the energy consumption of only the algorithm. Excluding the energy consumption from data loading, the energy conservation of the parallel program could be saved about 79.44%, greatly improving the battery working hours of smartphone.

**Table 7:** Power consumption for sequential and parallel algorithm on ECG data from 310 hospital

ECG no.	Data loading	The sequential		The parallel	
	Energy (mWh)	Power (mW)	Energy (mWh)	Power (mW)	Energy (mWh)
1	6.84	1908.98	35.08	1845.93	12.48
2	6.36	1861.02	30.52	1841.35	11.5
3	6.65	1819.40	35.49	1807.44	12.86
4	6.55	1800.24	35.32	1811.56	11.21
5	6.96	1893.36	35.40	1818.13	12.83
6	6.12	1900.71	32.14	1805.11	11.82
7	6.22	1842.94	33.55	1858.84	12.44
Average	6.53	1860.95 ± 42.17	33.93 ± 1.95	1826.91 ± 21.44	12.16 ± 0.65

## Conclusion

This paper proposed a parallel automatic ECG analysis algorithm based on smartphone GPUs. The processing steps of artifact removal, QRS complex detection, and ECG waveform classification were implemented in parallel according to time cost and parallel feasibility analysis of the sequential automatic ECG analysis algorithm. Compared with the sequential algorithm, the average time cost of the parallel automatic ECG analysis algorithm on the long-term ECG dataset from the 301 hospital was reduced from 7.018 ± 0.592 to 1.215 ± 01.40 s, and a speedup of 5.81 ± 0.39× was achieved. Additionally, the average power and execution time of the parallel automatic ECG analysis algorithm was less than that of the sequential automatic ECG analysis algorithm, and about 64.16% of the battery energy was saved. If the energy consumption from data loading was excluded, the parallel program could save 79.44% of the energy consumption.

The reduction of response time and battery energy consumption in ECG analysis not only bring better quality of experience to holter users, but also make it possible to use mobile devices as ECG terminals for healthcare professions such as physicians and health advisers, enabling them to inspect patient ECG recordings onsite efficiently without the need of a high-quality wide-area network (WAN) environment. This is very helpful to extend the use of onsite ECG monitoring in rural, outdoor or ambulatory situations. Moreover, the migration of ECG processing pipeline to mobile device without heavy CPU occupation paves the way for real-time identification of cardiac arrhythmias which is of great value in prevention of acute cardiovascular event (e.g., stroke and myocardial infractions). This would be the further direction of our research.
